# Treatment of Clavicle Fractures

**Published:** 2012-01-18

**Authors:** P Paladini, A Pellegrini, G Merolla, F Campi, G Porcellini

**Affiliations:** Unit of Shoulder and Elbow Surgery, D. Cervesi Hospital, Cattolica - Italy

## Abstract

Clavicle fractures are very common injuries in adults (2–5%) and children (10–15%) (1) and represent the 44–66% of all shoulder fractures (2). Despite the high frequency the choice of proper treatment is still a challenge for the orthopedic surgeon. With this review we wants to focus the attention on the basic epidemiology, anatomy, classification, evaluation and management of surgical treatments in relationship with the gravity of injuries. Both conservative and surgical management are possible, and surgeons must choose the most appropriate management modality according to the biologic age, functional demands, and type of lesion. We performed a review of the English literature thought PubMed to produce an evidence-based review of current concept and management of clavicle fracture. We finished taking a comparison with our survey in order to underline our direct experience.

## Introduction

Clavicle fractures are common injuries in adults (2–5%) and children (10–15%) (1) and represent the 44–66% of all shoulder fractures (2). Despite the high frequency the choice of proper treatment is still debated.

Criteria for conservative or surgical management are not clearly established; therefore the appropriate management of these fractures should consider several factors, mainly the patient’s biologic age, functional demands and the type of lesion. A search of the English articles published from 1968 to2011 in the National Library of Medicine database (Medline), PubMed and Embase was performed using the words “Fracture, Clavicle, Treatment” as subjects headings to produce an evidence-based review of the current concepts and management of clavicle fractures.

## Epidemiology

The incidence of clavicle fractures in adolescent and adult population is suggested to be between 29 and 64 per 100.000 persons (2–4). As usual, in many traumas, its prevalence is highest among the young population even if also shows a bimodal age distribution with a rate in females that overtake males after the sixth decade of life as a result of osteoporosis and differences in life expectancy. The mean age has been reported to be 29.3 years, and the incidence appears to decrease significantly after the second decade of life. Males are affected approximately twice as often as females (67.9% vs 32.1%). These injuries may also have a seasonal correlation, with one epidemiologic analysis noting an increase during the summer (3).

In adults, more than two-thirds of these injuries occur at the diaphysis of the clavicle, and these injuries are more likely to be displaced as compared with medial and lateral third fractures (probably due to the greater exposure to high energy trauma through sports and traffic accidents). In children, up to 90% of clavicle fractures are midshaft fractures (3;5). Lateral-third fractures are less common, accounting for approximately 25% of all clavicle fractures, and are less likely to be displaced than those occurring in the midshaft. Medial-third fractures comprise the remaining 2% to 3% of these injuries (1).

## Clinic anatomy

The clavicle is the first bone in the human body to begin intramembranous ossification directly from mesenchyme during the fifth week of fetal life. Similar to all long bones, the clavicle has both a medial and lateral epiphysis. The growth plates of the medial and lateral clavicular epiphyses do not fuse until the age of 25 years (2).

Peculiar among long bones is the clavicle’s S-shaped double curve, which is convex medially and concave laterally. This contouring allows the clavicle to serve as a strut for the upper extremity, while also protecting and allowing the passage of the axillary vessels and brachial plexus medially. The cross-sectional geometry also changes along its course. It progresses from more tubular medially to flat laterally. This change of contour, which is most acute at the junction of the middle and outer thirds, may explain the frequency of fractures seen in this area (8).

The lateral clavicle is anchored to the coracoid process by the coraco-clavicular ligament, composed of the lateral trapezoid and medial conoid parts. The static joint stabilizers are the AC ligaments, controlling the horizontal stability, and the CC ligament controlling the vertical stability. The dynamic stabilizers are the deltoid and trapezius muscles. The trapezius muscle attaches at the dorsal aspect of the acromion, part of the anterior deltoid muscle inserts on the clavicle medial to the AC joint. Their force vectors prevent excessive superior migration of the distal clavicle after disruption of the AC and CC ligaments alone (2).

The deltoid, trapezius, and pectoralis major muscles have important attachments to the clavicle. The deltoid muscle inserts onto the anterior surface of the lateral third of the clavicle, and the trapezius muscle onto the posterior aspect. The pectoralis major muscle inserts onto the anterior surface of the medial two thirds.

## Mechanism of injury

With the exception of the rare pathologic fracture due to metastatic or metabolic disease, clavicle fractures are typically due to trauma (2). Younger individuals often sustain these injuries by way of moderate to high-energy mechanisms such as motor vehicle accidents or sports injuries, whereas elderly individuals are more likely to sustain injuries because of the sequela of a low-energy fall (6). Although a fall onto an outstretched hand was traditionally considered the common mechanism, it has been found that the clavicle most often fails in direct compression from force applied directly to the shoulder. In a study of 122 consecutive patients, 87% clavicle injuries resulted from a fall onto the shoulder, 7% resulted from a direct blow, and 6% resulted from a fall onto an outstretched hand (7).

## Classification

A number of classification systems have been proposed to aid in the description of clavicle fracture patterns for clinical and research purposes (1) To date, most modern clavicle fracture classification systems are primarily descriptive and not predictive of outcome. The first widely accepted classification system for clavicle fractures was described by Allman (9) in 1967. Fractures were classified based on their anatomic location in descending order of fracture incidence. Type I fractures occur within the middle third of the clavicle, whereas type II and type III fractures represent involvement of the lateral and medial thirds, respectively.

Fractures of the lateral third of the clavicle were further sub classified by Neer, (10) recognizing the importance of the coraco-clavicular (CC) ligaments for the stability of the medial fracture segment. A type I lateral clavicle fracture occurs distal to the CC ligaments, resulting in a minimally displaced fracture that is typically stable. Type II injuries are characterized by a medial fragment that is discontinuous with the CC ligaments. In these cases, the medial fragment often exhibits vertical instability after loss of the ligamentous stability provided by the CC ligaments. Type III injuries are characterized by an intra-articular fracture of the acromio-clavicular joint with intact CC ligaments. Although these fractures are typically stable injuries, they may ultimately result in traumatic arthrosis of the acromio-clavicular joint. A more subtle fracture may require special radiographic views for identification and may be mistaken for a first-degree acromio-clavicular joint injury.

A more detailed classification system (Edinburgh classification) was proposed by Robinson (4). Similar to earlier descriptions, the primary classification is anatomically divided into medial (type I), middle (type II), and lateral (type III) thirds. Each of these types is then subdivided based on the magnitude of fracture fragment displacement. Fracture displacement of less than 100% characterizes subgroup A, whereas fractures displaced by more than 100% account for subgroup B. Type I (medial) and type III (lateral) fractures are further subdivided based on articular involvement. Subgroup 1 represents no articular involvement, and subgroup 2 is characterized by intra-articular extension. Similarly, type II (middle) fractures are sub-categorized by the degree of fracture comminution. Simple or wedge-type fracture patterns make up subgroup 1, and comminuted or segmental fracture patterns represent subgroup 2.

Craig (11) further modified Neer type II lateral clavicle fractures by stressing the importance of the conoid ligament and separately classifying intra-articular and pediatric clavicle fractures. A recent comparison of these classification systems showed that Craig’s classification was most prognostic when predicting delayed union or nonunion of lateral-third fractures and Robinson’s classification had the greatest prognostic value for middle third fractures (11; 12).

## Evaluation

Individuals with clavicle fractures will almost uniformly report an episode of trauma that has resulted in acute shoulder pain (2). Determining the mechanism is critical; while simple falls often produce isolated fractures, the high-energy mechanisms seen in the younger population can produce associated rib, scapular, or ipsilateral upper extremity fractures (2). Additionally, pneumothorax, hemothorax, and nerve and vascular injury have all been reported in association with clavicle fractures (13). One should also ask whether there have been previous injuries to the ipsilateral clavicle and determine the patient’s hand dominance, as these factors may alter the treatment decision.

On examination, ecchymosis and a prominence over the fracture site may be observed. Skin breaks or skin tenting must be identified, as both are indications for emergent operative treatment. Palpation along the subcutaneous border of the bone should reveal an area of tenderness and potential step-off of the normally smooth contour. Attempted range or motion of the shoulder will be limited and produce pain and even palpable crepitus. We typically defer a thorough range-of-motion examination at the initial visit. A neurovascular examination is essential. Motor and sensory function of the radial, ulnar, median, and axillary nerves should be confirmed. The radial pulse should be palpated and capillary refill compared with the contralateral side.

Additional work-up should consist of a minimum of 2 radiographic views. A standard AP view and a serendipity view (aimed 30°–45° cephalad) should be reviewed to determine fracture pattern, degree of displacement, and rule-out pneumothorax. Additionally, many orthopedists believe that a clavicle fracture is evidence of enough shoulder traumas to justify obtaining a full shoulder series, consisting of an AP, scapular “Y”, and axillary lateral view. For Allman Group II (lateral) fractures, an axillary view should be obtained to determine if there is AP displacement of the fracture fragments. Additionally, if there is a question regarding disruption of the CC ligaments, a weighted view can easily be obtained at the time of initial radiographs. A computed tomography (CT) scan may be required to determine the direction of displacement of Group III (medial) fractures, as posterior displacement risks injury to underlying neurovascular structures. Computed tomography scanning may also be helpful in the setting of nonunion or malunion, but are not typically a part of the initial evaluation.

## Conservative treatment

Conservative or non-surgical treatment is the norm for middle-third clavicle fractures, and is recommended for not displaced fractures (14) given the generally low incidence of non-union after conservative treatment of these fractures with rates ranging from 0.03% to 5.9% (14–16). There are numerous conservative treatment options available, the most common being the use of a sling or ’figure-of-eight’ bandage (also known as figure-of-eight splint, or back-pack bandage), or a combination of these two methods (17–18). There appears to be no consensus on the optimal duration of immobilization; some have recommended two to six weeks (13; 18–19). Often no subsequent therapy is suggested to the patient. Sometimes, however, a patient will require stretching exercises to regain motion. We prefer to follow the patient with a structured rehabilitation in order to have a satisfactory outcome for most patients. To protect the healing clavicle, it is important to avoid contact sports for a minimum of 4 to 5 months (20).

Recent studies on displaced midshaft clavicular fractures indicate a significant unmet medical need, with non-union rates of 15% and unsatisfactory patient-reported outcomes in around a third of patients (15;21). These findings have prompted a recent increase in surgical fixation of displaced fractures. The comparison of surgery versus conservative treatment is the subject of a forthcoming Cochrane review (22).

## Surgical treatment

**Fig 1:**
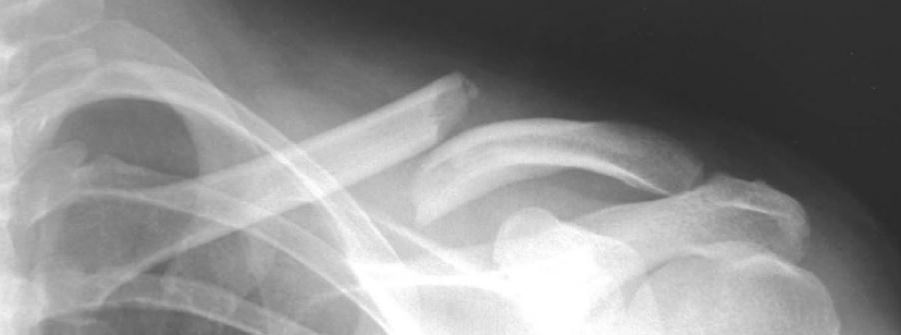
Xray shows a displaced middle third fracture of clavicle.

**Fig 2:**
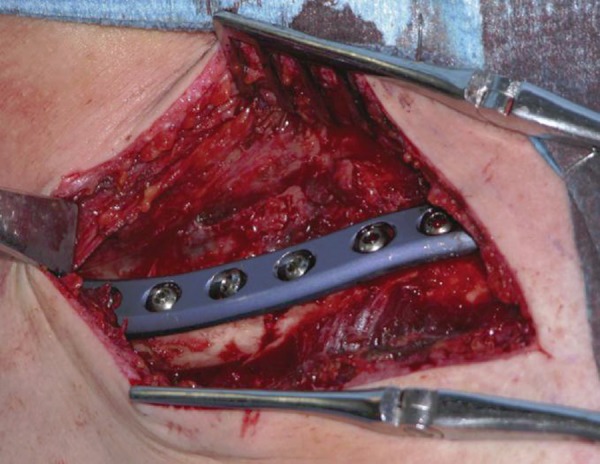
ORIF with plate and screws of displaced middle third fracture of clavicle.

**Fig 3:**
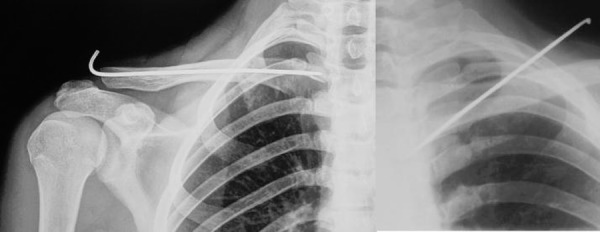
Xray shows a K-wire fixation on middle third clavicle fracture and the migration of K-wire.

Different surgical treatments are reported in literature liked by different type of fractures and injury. Surgical treatment of medial-end clavicle fractures is indicated if mediastinal structures are placed at risk because of fracture displacement, in case of soft-tissue compromise, or when multiple trauma and/or “floating shoulder” injuries are present (1). Closed or open reduction should be performed to reduce the displaced fragment in an emergent fashion. (23–24). When open reduction is necessary, several techniques have been described for internal fixation of fracture fragments. These include wire or plate fixation (Fig. 1 and Fig. 2) and interosseous sutures. (23–25) In general, Kirschner wire fixation has proven unsafe because of breakage and migration (Fig. 3). By contrast, use of interosseous wires or suture and modified hooked Balser plate fixation appears more successful but requires a second operation for hardware removal (23–25). Most injuries in children and adolescents involving the medial end of the clavicle consist of epiphyseal separations. This is because the medial epiphysis of the clavicle does not ossify until age 20 years and ossification centers rarely fuse before age 25 years (26). It is important, however, to differentiate epiphyseal separations from true sterno-clavicular joint dislocations because of the remodeling potential and because the treatment of these 2 diagnoses can differ greatly. A computed tomography scan can be helpful to distinguish these entities (24;26).

About the middle third clavicle fractures definitive indications for acute surgical intervention include skin tenting, open fractures, the presence of neurovascular compromise, multiple trauma, or floating shoulder. Outside of these indications, the management of displaced fractures of the midshaft (Edinburgh type 2B) remains somewhat controversial. Recent literature is challenging the traditional belief that midshaft clavicle fractures uniformly heal without functional deficit. This paradigm shift is supported by several prospective studies by members of the Canadian Orthopedic Trauma Society, who reported higher nonunion rates and functional deficits after nonsurgical treatment of midshaft clavicle fractures when compared with internal fixation (21;27–28). Other authors suggest that specific clavicle fracture types are at higher risk for poor patient-reported outcomes (16). To this end, a retrospective series of 52 non-operatively treated patients showed that displaced fractures with shortening of 2 cm or more are predictive of higher nonunion or symptomatic malunion rates (29). Other studies have shown that nonunion rates may be as high as 20% in displaced and comminuted fractures after nonsurgical treatment and that strength and endurance deficits are more common in these cases (4;30). These reports, in combination with a more prognostic classification system, have led many authors to recommend acute surgical fixation for these fracture subtypes (14).

Therefore, relative indications for acute surgical treatment may include younger, active patients with clavicle shortening greater than 1.5 to 2 cm, significant cosmetic deformity, or multiple-trauma situations. Under these auspices, surgical fixation may provide more optimal outcomes and earlier return to sport. Adequate counseling regarding the risks, benefits, and likely results of treatment should occur in these circumstances. Late intervention should be considered for persistently symptomatic nonunion or malunion or if acromio-clavicular arthritic changes occur.

Open reduction and internal fixation of clavicle fractures can be performed with either plate or intramedullary pin fixation. Plate fixation can provide immediate rigid fixation, helping to facilitate early mobilization (10;29;31–32). However, it is thought that superior clavicle plating may result in a greater risk to underlying neurovascular structures and may be more prominent than anterior plating or intra-medullary pin fixation (16;33). A study by Bostman et al (34) reported that complication and reoperation rates may be as high as 43% and 14%, respectively, if hardware removal is considered. Other reported complications include infection, hardware failure, and hypertrophic scarring (34). The recent introduction of anatomically contoured clavicle plates may reduce the need for hardware removal (27).

Antegrade or retrograde intramedullary pin fixation is typically a more cosmetic technique, requiring a smaller incision and less stripping of the clavicle compared with plate fixation. Intramedullary pins frequently cannot be statically locked, thereby providing less rotational and length stability compared with other fixation techniques (35–38). The intramedullary pin also requires routine removal after clinical and radiographic evidence of healing. Reported complications of this specific technique include implant breakage, skin breakdown, and temporary brachial plexus palsy (39–41). A recent study reported major complications requiring revision surgery in 5 of 58 analyzed patients (40). All revisions were performed for fracture nonunion.

Reported outcomes of surgical treatment of midshaft clavicle fractures have become more favorable over the past 2 decades. A meta-analysis of current data on not displaced fractures suggested a relative risk reduction of 72% and 57% for nonunion as compared with nonoperative treatment by use of intramedullary pin fixation and plate fixation, respectively (16). For displaced fractures, the relative risk reduction increased to 87% and 86%, respectively.

Patient-reported satisfaction scores may also be superior with early surgical management in some circumstances. A multicenter trial reported better functional outcomes, lower malunion and nonunion rates, and a shorter overall time to union in operatively treated clavicle fractures after plate fixation (27). A significant improvement in functional outcome scores was also reported when operatively and non- operatively treated fractures were compared.

The indication for surgical treatment of lateral-third clavicle fractures is based on the stability of the fracture segments, displacement, and patient age. The integrity of the CC ligaments plays a key role in providing stability to the medial fracture fragment. Displacement of the medial clavicle is seen when the CC ligaments are disrupted (Edinburgh type 3B). It is established that this fracture configuration leads to nonunion rates as high as 28% (4;10). Other authors have reported that the risk of nonunion increases with advancing age and displacement (14;42–43). Again, the presence of soft-tissue compromise, multiple traumas, and floating shoulder are also indications for operative treatment.

Many surgical techniques have been proposed for fixation of lateral-end fractures. These include Kirschner wire fixation (44), CC screws (45), plate or hook-plate fixation (46–47), and suture and sling techniques (47–50). However, reported complication rates limit their utility. For example, migration rates of up to 50% and failure of Kirschner wire fixation have led several authors to recommend that it not be used as a primary fixation technique (43;51–52).

Furthermore, the use of CC screw fixation is limited by the fracture location and extent of comminution. In addition, screws must be routinely removed because they can limit shoulder girdle motion. Some failures noted in patients treated with CC screw fixation are likely due to the combination of rigid (screw) fixation and the motion normally present at this location.

Plate fixation can also be used in circumstances where the distal fragment allows sufficient fixation (42). A hook plate might be indicated if the distal fragment is inadequate for screw placement. This is performed in a fashion similar to standard plate fixation with the exception that distal fixation is achieved by placing the “hooked” end of the implant under the acromion to maintain a satisfactory reduction.

Finally, suture and graft sling techniques can be used to reconstruct CC ligaments in a manner similar to anatomic acromio-clavicular joint reconstruction. These techniques can be used to reinforce other fixation techniques or as the primary mode of reconstruction (47–50).

Nonoperative management of lateral clavicle fractures results in a good outcome in up to 98% of minimally displaced or not displaced fractures (14) while rates increase with displacing of the fractures (4;10;53).

The timing of surgery for lateral-end fractures seems more important for patient outcome when compared with medial-third fractures (42). Although the union rate does not seem to be influenced by acute or delayed treatment, the complication rate may be higher when the surgical treatment is delayed (7% vs 36%) (42). Lateral clavicle fractures that exhibit intra-articular extension may result in an increased risk of acromio-clavicular joint degeneration. If acromio-clavicular arthritis occurs, the patient may require a late distal clavicle excision. Despite the limitations of CC screw fixation, the results of fracture healing and restoration of shoulder function are mostly favorable, although only small cohorts have been reported (54–55). Plates have also been used successfully, but complications such as peri-implant fracture, nonunion, stiffness, and arthritic progression are of concern in up to 15% of patients (42;51;56). Finally, acceptable functional results and high union rates have been reported with the use of suture or graft sling techniques to reconstruct CC ligaments (47–50).

## Complications

Complications of clavicle fracture include radiographic and symptomatic malunion and shoulder deformity, non-union and infections.

Displaced and nonoperative treated clavicle fractures all heal with some degree of malunion secondary to angulation and shortening (2;57). Although malunion is commonly asymptomatic and has traditionally been described as a pure cosmetic concern, recent studies have shown that functional limitations do occur (58). Clavicular shortening of > 15 mm has been associated with shoulder discomfort and dysfunction and can change shoulder dynamics (58–60). Malunion may also be symptomatic, producing pain, neurovascular compromise, and upper extremity weakness (21;61). For these patients, late corrective osteotomy and plate fixation with bone grafting has been shown to improve symptoms related to their malunion (58;62). It should be stressed, however, that clinically symptomatic malunion, not asymptomatic radiographic malunion, is the indication for operative intervention. ). Nonunion rates, however, are much greater for displaced fractures (Neer type II and Edinburgh type 3B) and are reported to be as high as 33% if treated nonoperative (4;10;53).The rate of nonunion following midshaft clavicle fractures has been reported to range from < 1% to 15% for displaced fractures (61). The rate of nonunion following nonoperative treated distal clavicle fractures is higher, and in the literature ranges from 11% to 40% in small case series (61), though not all radiographic nonunion are symptomatic. Risk factors for nonunion include female sex, older age, degree of displacement, and comminution (14). Symptomatically, distal and shaft nonunion are similar and are associated with pain, restriction of shoulder movement, weakness, and neurovascular symptoms, including thoracic outlet syndrome and subclavian vein compression (61). However, in elderly individuals, nonunion of type II fractures may be associated with minimal symptoms and high patient satisfaction. Therefore, nonoperative treatment may still be considered even in light of high nonunion rates (14;53;61). Plate fixation is the primary treatment for symptomatic nonunion of a clavicle shaft fracture. In the setting of hypertrophic nonunion, increased stabilization with ORIF may be all that is required. In addition to plate fixation, in the setting of atrophic nonunion bone grafting (often from the iliac crest), it has been shown to decrease time to union and restore length (31;63). Treatment options for nonunion of a distal clavicle fracture depend on the size of the distal fragment: if the fragment is small and the CC ligaments are intact, distal fragment excision is recommended; however, if the distal fragment is large enough, internal fixation has been shown to be effective in promoting healing (61). Methods of internal fixation for nonunion of distal clavicle fractures are similar to primary operative treatment of distal clavicle fractures, as described previously.

As with any surgical procedure, infection and wound dehiscence are reported complications of clavicle ORIF. As the clavicle is subcutaneous, the soft tissue envelope available for closure over implanted hardware is relatively thin, likely contributing to rates of wound complications. In a recent randomized trial, there was a wound complication rate of approximately 5% (27); however, all patients were successfully managed with local wound care, antibiotics, and hardware removal after fracture union. Of note, infection with propionibacterium acnes common about the shoulder, as compared with other surgical sites, and this organism should be covered empirically during treatment, especially as it is slow growing, and standard cultures may remain negative for some time.

Although plating of the clavicle spans the original fracture site, it rarely involves fixation along its entire length. Re-fracture secondary to additional trauma either medial or lateral to the original hardware is thus possible, and in fact is reported at rate of between 1% and 2% (27). Re-fracture necessitates revision ORIF.

Due to the limited soft tissue envelope, the plating used for ORIF can be prominent, especially in thin individuals. Positioning the hardware along the anterior surface of the clavicle, as opposed to the more traditional superior position, may reduce the rates of hardware irritation, which is often caused by backpacks or bra straps. The rates of removal of hardware for prominent hardware are reported to be around 8% (27).

## Unpublished data

**Fig 4:**
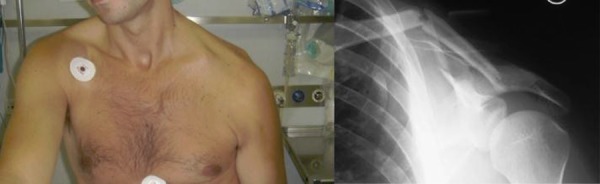
Clinical and Xray images of floating shoulder.

**Fig 5:**
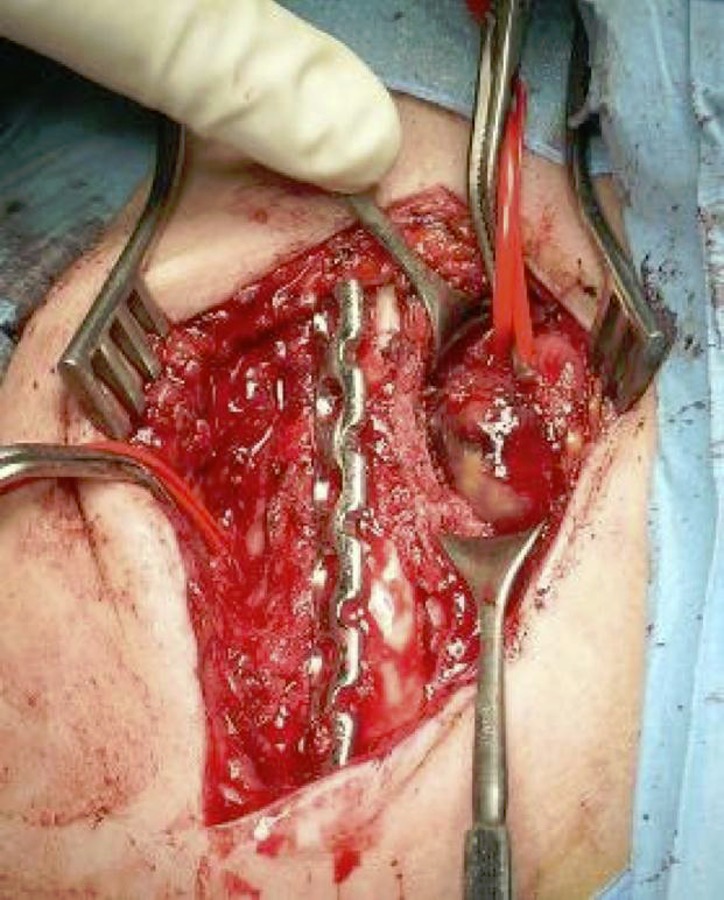
Intraoperative view of pseudo-aneurism of subclavian artery.

**Fig 6:**
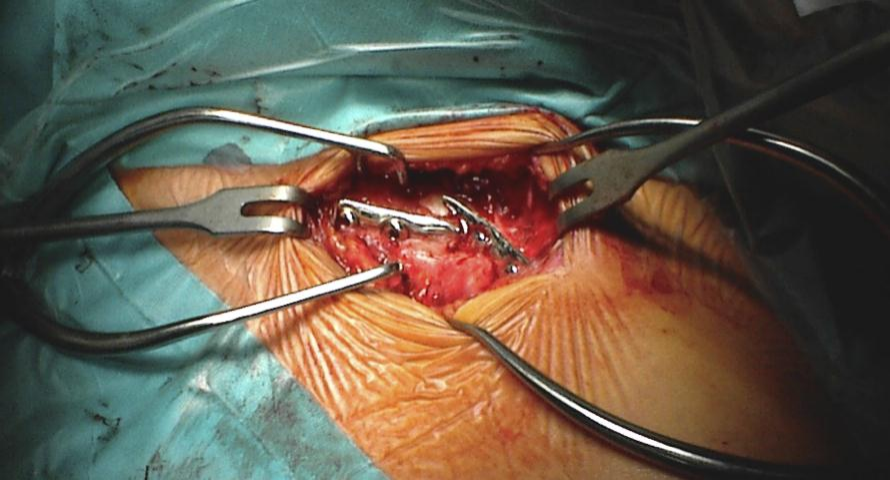
Revision in ORIF of re-fracture involving the plate of previous fixation.

From 1994 to 2009 in our Unit 63 patients were surgically treated for displaced clavicle fractures (M/F: 39/28; mean age: 36 years old; min 18 - max 59 years). Four patients presented a floating shoulder (Fig. 4). 61 to 67 had an excellent score on clinical evaluation with Constant score at 24 months of follow up. 6 patients had surgical complications. We had a very unusual case of a vascular complication, pseudo-aneurism of subclavian artery, in a patient treated with plate and screws (Fig. 5). Three cases of infection, 1 on a patient treated with K-wire reduction and fixation, 2 cases of plate mobilization and 1 case of plate rupture (Fig. 6).

## Conclusion

The treatment of the clavicle fractures is still controversial and debated. The use of plate and screws fixation represents the gold standard in displaced and comminuted fractures. Non-operative treatment is mandatory in not displaced cases.
